# Transcriptome and Metabolome-Based Analysis Reveals the Molecular Mechanisms Underlying the Differences in Tanshinone and Salvianolic Acid Content Between *Salvia miltiorrhiza* Roots and Leaves

**DOI:** 10.3390/genes17030280

**Published:** 2026-02-27

**Authors:** Fawang Liu, Yingying Xu, Lei Pan, Yin Zhang, Mengping Ding

**Affiliations:** 1 School of Biological and Food Engineering, Engineering Research Center for Development and High Value Utilization of Genuine Medicinal Materials in North Anhui Province, Suzhou University, Suzhou 234000, China; yuebing0514@163.com (Y.X.); 18956600948@163.com (L.P.); zydzh_2026@163.com (Y.Z.); 13695741106@163.com (M.D.); 2 Anhui Engineering Laboratory for Medicinal and Food Homologous Natural Resources Exploration, Hefei Normal University, Hefei 230601, China

**Keywords:** *Salvia miltiorrhiza*, metabolic and transcriptional, secondary metabolism, tanshinone and salvianolic acid biosynthesis

## Abstract

**Background/Objectives: ***Salvia miltiorrhiza* (Danshen) is a well-known medicinal herb in traditional Chinese medicine. It produces tanshinones and salvianolic acids as key bioactive constituents in its roots, yet the molecular basis for their tissue-specific accumulation is still poorly understood. This study aims to identify candidate functional genes involved in the biosynthesis of tanshinones and salvianolic acids, and to reveal the molecular basis underlying their tissue-specific accumulation in *S. miltiorrhiza*. **Methods:** For this purpose, we compared transcriptomic and metabolomic differences between the roots and leaves, and further measured a set of physiological parameters, including the POD, SOD, CAT and PAL activities, as well as the total phenols and flavones contents. **Results:** Metabolomic analysis identified 6805 metabolites, of which 241 were differentially accumulated between roots and leaves, with 172 upregulated in roots. The elevated metabolites included gibberellin, cryptotanshinone, decursinol angelate, chalcone, and psoralenol. Transcriptome analysis identified 32,700 annotated genes, with 9895 showing differential expression between roots and leaves, including 4199 upregulated in roots. Roots exhibited higher levels of phenols and flavones, as well as significantly greater POD, SOD, CAT, and PAL activities. **Conclusions:** Integrated omics analysis identified putative candidate genes including *CPS*, *KS*, and *P450s* as potential contributors for tanshinone and salvianolic acid biosynthesis. The identified genes provide valuable resources for molecular breeding, offering opportunities to improve the medicinal quality of *S. miltiorrhiza*.

## 1. Introduction

*S*. *miltiorrhiza* Bunge is a medicinal plant belonging to the *Lamiaceae* family. Its roots have been widely used in traditional Chinese medicine for anti-tumor activity, improved circulation, menstrual regulation, pain relief, and anxiety relief [1]. *S. miltiorrhiza* contains two major classes of bioactive compounds: fat-soluble diterpenes (tanshinones), and water-soluble phenolic acids; these two classes of compounds accumulate predominantly in the root of *S. miltiorrhiza*, contributing to its high medicinal value [2,3]. Tanshinones are predominantly synthesized in the roots of *S. miltiorrhiza*; to date, over 50 tanshinone-related compounds and more than 30 salvianolic acid analogs have been identified [4]. Tanshinone biosynthesis is part of the diterpenoid metabolic pathway, while salvianolic acid originates from the phenylpropanoid pathway through the condensation of tyrosine- and phenylalanine-derived precursors. Multiple P450s are involved in modifying both tanshinone and salvianolic acid, but many downstream biosynthetic steps remain unknown [5,6,7,8,9].

The P450s superfamily plays crucial roles in the biosynthesis of terpenoids. In *S. miltiorrhiza*, 116 *P450* genes have been annotated, among them, several genes (including *SmCYP76AH1*, *SmCYP76AH3*, *SmCYP76AK1*, *SmCYP71D373*, and *SmCYP71D375*) have been functionally validated to be associated with tanshinone biosynthesis. However, multiple oxidation steps in the pathway are still unassigned, suggesting the involvement of additional, yet-to-be-characterized P450s [10,11,12]. Similarly, the upstream pathway leading to rosmarinic acid, the direct precursor of salvianolic acid, is well established, involving key enzymes such as phenylalanine ammonia-lyase (PAL), cinnamate 4-hydroxylase (C4H), 4-coumarate-CoA ligase (4CL), tyrosine aminotransferase (TAT), and rosmarinic acid synthase (RAS). However, the enzymatic steps converting rosmarinic acid into salvianolic acid derivatives remain poorly understood, and the corresponding genes require further identification and validation.

*S. miltiorrhiza* roots are widely used in medicine due to their high accumulation of tanshinones and salvianolic acids, but the molecular basis for this tissue-specific metabolite distribution remains unclear. Previous studies have identified key genes involved in tanshinones and salvianolic acids biosynthesis, but their regulatory mechanism in tissue-specific accumulation remains largely elusive. Therefore, there is an urgent need to identify and characterize the *P450s* involved in tanshinone modification, elucidate the biosynthetic pathway from rosmarinic acid to salvianolic acid, and understand the regulatory mechanisms governing tissue-specific accumulation of tanshinones and salvianolic acids through integrative omics approaches.

In this study, we performed a comprehensive comparative analysis of secondary metabolite accumulation and gene expression profiles between aerial (leaves) and underground (roots) tissues of *S. miltiorrhiza* using integrated transcriptomic and broad-targeted metabolomic strategies. Our results showed that tanshinones and phenolic acid derivatives were enriched in roots. Key biosynthetic genes, including *CPS* and *KS* in the tanshinone biosynthesis pathway, and *PAL*, *TAT*, and *4CL* in the salvianolic acid biosynthesis pathway, were significantly upregulated in roots. Furthermore, co-expression network analysis identified multiple candidate P450s and downstream modifying enzymes potentially involved in the oxidation of tanshinone intermediates and the transformation of salvianolic acid precursors. Collectively, this work provides critical insights into the potential molecular mechanisms underlying tissue-specific accumulation of tanshinones and phenolic acids in *S. miltiorrhiza*, and proposes a preliminary working model for the biosynthesis of these bioactive compounds.

## 2. Materials and Methods

### 2.1. Plant Materials

Three-year-old *S. miltiorrhiza* plants were originally collected from the Yongqiao District of Suzhou City, Anhui Province, China (33°38′24″ N, 116°58′38″ E). These plants were cultivated at the Engineering Research Center for Development and High-Value Utilization of Genuine Medicinal Materials. The plants were grown in humus-rich soil under a temperature of 24 °C, a photosynthetic photon flux density of 300 μmol·m^−2^·s^−1^, a 16 h/8 h light/dark photoperiod, and a relative humidity of 55%. The leaves and roots were separately harvested from three individual *S. miltiorrhiza* and immediately frozen in liquid N2. For the downstream assays, 0.1 g of fresh tissue was used for physiological assays, while 0.05 g was used for combined transcriptomic and broad-targeted metabolomic assays.

### 2.2. Measurement of Physiological and Biochemical Indicators

Enzyme activity assays were performed using crude protein extracts, while total phenolics and flavonoids were extracted according to the manufacturer’s instructions (Solarbio, Beijing, China). We selected phenylalanine ammonia-lyase (PAL), a key rate-limiting enzyme in the salvianolic acid biosynthetic pathway closely associated with tissue-specific phenolic acid accumulation, and peroxidase (POD), superoxide dismutase (SOD), and catalase (CAT), representative antioxidant enzymes involved in regulating redox homeostasis that contribute to the tissue-specific accumulation of secondary metabolites in *S. miltiorrhiza*. The activities of the enzymes were measured using a ReadMax 1200 microplate reader (Flash, Shanghai, China) across full-wavelength absorbance [13,14,15,16].

To determine POD activity, 15 μL of crude protein extract was added to 650 μL of 2% guaiacol, 270 μL of PBS buffer, and 135 μL of hydrogen peroxide. The mixture was then mixed and the absorbance at 470 nm was recorded at 0 min and 1 min to calculate the change in absorbance values (△A) [14,15]. POD activity was calculated using the following formula:

(1) $$POD\;(U/g\;FW)=\frac{7133\times △A}{FW}$$

Similarly, SOD activity was measured using UV-visible spectrophotometry at 560 nm. The percentage of inhibition of formazan synthesis was then calculated (% inhibition) [16]. Inhibition and SOD activity was calculated using the following formula:

(2) $$\%\;Inhibition=\left(\frac{{△A}_{blank}-{△A}_{sample}}{{△A}_{blank}}\right)\times 100\%$$

(3) $$SOD\;(U/g\;FW)=\frac{10\times \%\;Inhibition}{1-\%\;Inhibition}÷FW$$

PAL activity was determined by monitoring the formation of *trans*-cinnamic acid at 290 nm. In brief, the reaction mixture contained L-phenylalanine and crude protein extract in borate buffer (pH 8.8), and was incubated at 40 °C for 1 h. The reaction was stopped with 6 mol/L HCl, and the absorbance change was measured [14,15]. PAL activity was calculated as follows:

(4) $$PAL\;(U/g\;FW)=\frac{17.3\;\times △A}{FW}$$

CAT activity was determined by measuring the decomposition of H_2_O_2_ at 240 nm. The reaction included crude enzyme extract and 0.3% H_2_O_2_ in PBS buffer [14,15]. The decrease in absorbance in 1 min was recorded, and CAT activity was calculated as follows:

(5) $$CAT\;(U/g\;FW)=\frac{459\;\times △A}{FW}$$

TP content was quantified using the tungstophosphoric acid (Folin–Ciocalteu) method. The phenolic compounds reduced the Folin–Ciocalteu reagent to produce a blue complex with maximum absorption at 760 nm. Gallic acid was used as the standard (linear range: 0.0024–0.156 mg/mL). By establishing standard curves and with detected absorption at 760 nm, the product’s content (X) was calculated [13]. The TP content was calculated as follows:

(6) $$TP\;(mg/g\;FW)=\frac{2.5\times X}{FW}$$

Similarly, TF content was determined using the aluminum chloride colorimetric method. Rutin was used as the standard (linear range: 0.0097–1.75 mg/mL). Flavone forms a red complex with Al^3+^ ions, which exhibits strong absorbance at 470 nm. By establishing standard curves, with detected absorption at 470 nm, the product’s content (X) was calculated [13]. The TF content was calculated as follows:

(7) $$TF\;(mg/g\;FW)=\frac{X}{FW}$$

All physiological and biochemical data were subjected to FDR (False Discovery Rate) correction for multiple testing, consistent with the statistical method used for the transcriptome analysis. The effect sizes and statistical uncertainties (mean ± SD) of all of the measured indicators were reported for three biological replicates.

### 2.3. Transcriptome Sequencing

Total RNA was extracted using the cetyltrimethylammonium bromide (CTAB) method [17]. The purity and concentration of isolated RNA were assessed using a NanoDrop 2000 spectrophotometer (Thermo Fisher Scientific, Waltham, MA, USA), with samples exhibiting an absorbance (A) ratio A260/280 ≥ 1.8, A260/230 ≥ 2.0 considered suitable for downstream applications. RNA integrity was assessed using an Agilent 2100-LabChip GX system (Agilent Technologies, Santa Clara, CA, USA), and only samples with an RNA quality number (RQN) ≥ 6.5 were selected for complementary DNA (cDNA) library construction. The cDNA library was prepared by Shanghai Majorbio Bio-pharm Biotechnology Co., Ltd. (Shanghai, China), following the standard Illumina protocol (Illumina, San Diego, CA, USA) [18].

After constructing the library, we used a Qubit 3.0 fluorescence quantification instrument for preliminary quantification and an Illumina NovaSeq X Plus platform for sequencing to get raw sequencing data, and further filtered using fastP (version 0.19.5) to yield clean reads. Raw reads were filtered with strict uniform thresholds, adapter sequences were removed, and low-quality reads (Qphred < 20) and short reads (<50 bp) were discarded; finally, only reads with Q30 ≥ 90% were retained as clean reads [19]. The clean reads were aligned to the reference genome of *S. miltiorrhiza* (available at https://ngdc.cncb.ac.cn/gwh/Assembly/10381/show, accessed on 4 July 2023) using HISAT2 (version 2.2.1) software to determine the genomic mapping positions [20]. The clean reads were also assembled using StringTie (version 11.0) in a reference-based approach [21]. To assess sample reproducibility and overall variance, clustering analysis and principal component analysis (PCA) were conducted using the Majorbio Cloud Platform (https://cloud.majorbio.com/page/tools/, accessed on 14 July 2023) by complete linkage method. Gene expression levels were normalized and reported as FPKM. Differential expression gene (DEGs) analysis was carried out using DESeq2 (version 1.10.1) by the Benjamini–Hochberg (BH) method [22], with the DEGs defined by the adjusted *p* < 0.05 and |log_2_(FC)| ≥ 1.

### 2.4. Metabolomics Analysis

Metabolomic profiling of *S. miltiorrhiza* was conducted by Oebiotech Co., Ltd. (Shanghai, China) using a method consistent with our previous studies [23]. Chromatographic separation was performed using a Waters ACQUITY UPLC I-Class coupled to a Xevo TQ-S MS/MS mass spectrometer (Waters Corporation, Milford, MA, USA) equipped with an electrospray ionization (ESI) source. Metabolites were separated on an ACQUITY UPLC HSS-T3 column (100 mm × 2.1 mm, 1.8 μm particle size). Mobile phase: 0.1% formic acid in water (A) and 0.1% formic acid in acetonitrile (B), gradient elution: 0–5 min, 5% B; 5–15 min, 5–95%B; flow rate: 0.3 mL/min). The mass spectrometry data were acquired in both positive and negative ionization modes. Compounds were identified using univariate statistical analysis (*t*-test) combined with multivariate statistical analysis (OPLS-DA/PLS-DA) and fold change value (FC), by matching accurate mass, retention time, and fragmentation patterns against HMDB, LipidMaps, and METLIN databases (accessed on 2 April 2024). The metabolites with *m*/*z* tolerance ≤ 5 ppm and an MS/MS spectral similarity score ≥ 0.8 were considered identical to the corresponding compounds in the databases. The differential metabolites (DEMs) were identified based on the criteria of adjusted *p*-value < 0.05 and Variable Importance in Projection (VIP) > 1.

### 2.5. Statistical Analysis

The enzyme activities were analyzed using GraphPad Prism (version 8.0.2) and Microsoft Excel (version 14). Data are expressed as mean ± SD from at least three biological replicates. Statistical significance between roots and leaves was assessed by one-way analysis of variance (ANOVA) followed by Duncan’s multiple range test (*p* < 0.05). Protein–protein interaction (PPI) networks were visualized using Cytoscape software (confidence score > 0.7, version 3.10.4) [24]. All statistical methods and parameters were unified across transcriptomic, metabolomic, and physiological data analysis to ensure consistency.

## 3. Results

### 3.1. Physiological Data Analysis of S. miltiorrhiza

**Figure 1:**
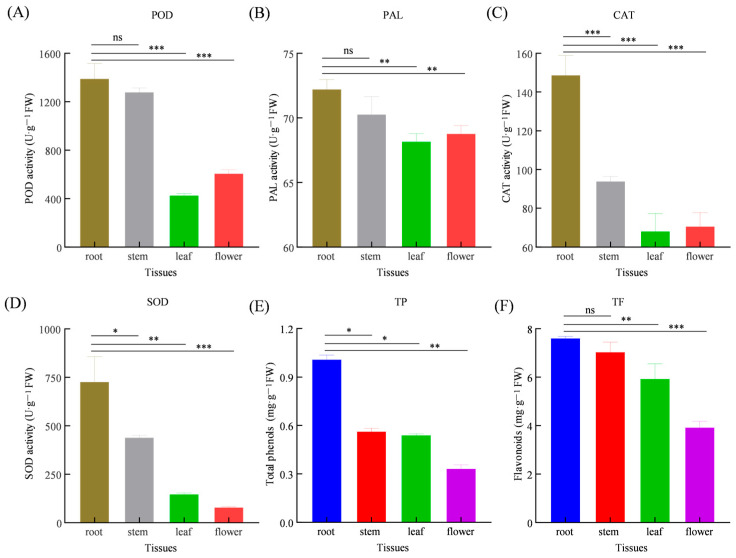
Antioxidant enzyme activities and total phenol (TP) and flavone (TF) contents in root, stem, leaf, and flower of *S. miltiorrhiza*. (**A**) POD activity; (**B**) PAL activity; (**C**) CAT activity; (**D**) SOD activity; (**E**) TP content; (**F**) TF content. Bars represent mean ± standard deviation (SD) from three biological replicates. ns: not significant; *: *p* < 0.05; **: *p* < 0.01; ***: *p* < 0.001.

The roots of *S. miltiorrhiza* showed significantly higher levels of TP and TF content compared to the leaves. The TP content in roots was 1.0091 ± 0.02 mg/g fresh weight (FW), followed by the stem (0.5637 mg/g ± 0.02 FW), leaf (0.5403 ± 0.01 mg/g FW), and flower (0.3332 ± 0.02 mg/g FW). Similarly, the TF was highest in the roots (7.6090 mg/g ± 0.06 FW) and lowest in the flowers (3.9320 ± 0.21 mg/g FW), indicating a strong tissue-specific pattern of secondary metabolite distribution. Antioxidant enzyme activities showed that, in the underground tissues, the POD, SOD, CAT, and PAL activities were 1394.26 ± 99.33 U/g, 728.75 ± 103.96 U/g, 148.87 ± 8.15 U/g and 72.24 ± 0.59 U/g, respectively. In contrast, the leaves and flowers exhibited lower and more similar activity levels, with the POD, SOD, CAT, and PAL activities ranging from 1282.51 to 430.01 U/g, 441.48 to 80.92 U/g, 94.09 to 68.39 U/g, and 70.30 to 68.19 U/g (Figure 1). These activity differences reflect the inherent developmental, structural, and functional specialization of different tissues.

### 3.2. Transcriptome Data Evaluation

**Figure 2:**
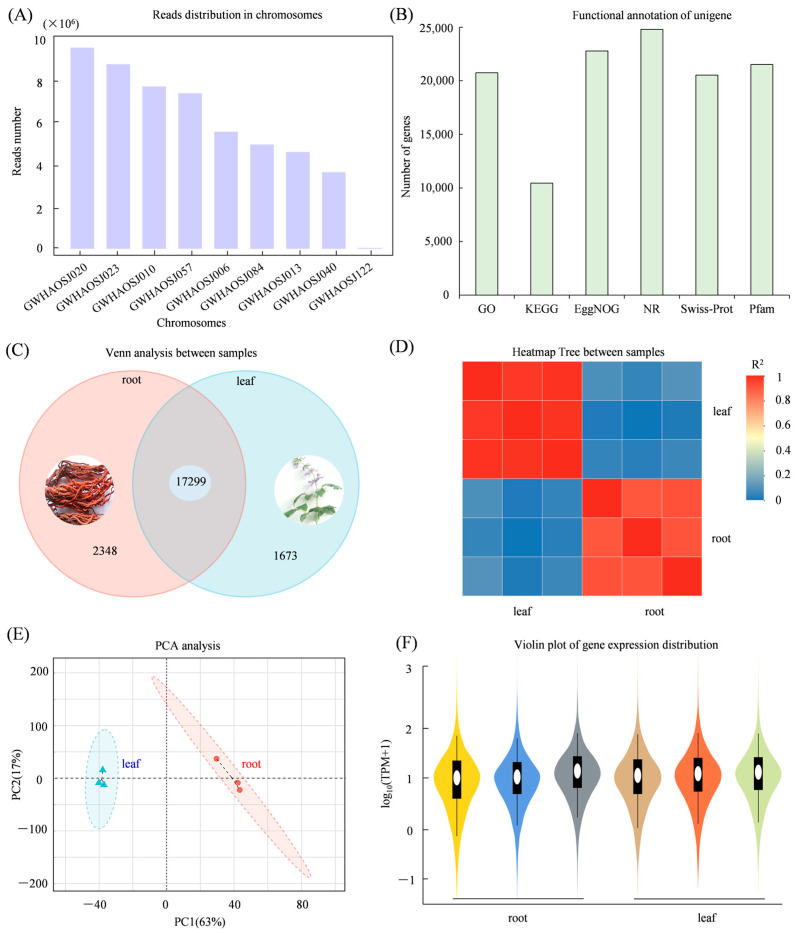
General overview and quality assessment of *S. miltiorrhiza* transcriptome data. (**A**) Chromosomal distribution of sequenced genes; (**B**) functional annotation results in NR, GO, KEGG, EggNOG, Swiss-Prot, and Pfam databases; (**C**) Venn diagram showing shared and unique genes between roots and leaves; (**D**) correlation analysis assessing reproducibility among root and leaf samples; (**E**) PCA distinguishing transcriptome profiles of roots and leaves; (**F**) gene expression distribution shown using violin plots.

Transcriptome sequencing resulted in a total of 54 Gb of high-quality clean data (NCBI SRA number PRJNA1284027), with a Q30 base percentage ≥ 92%. The clean reads were aligned to the *S. miltiorrhiza* reference genome (accession: GWHAOSJ00000000; https://ngdc.cncb.ac.cn/gwh/Assembly/10381/show, accessed on 4 July 2023). Over 92% of the mapped reads were uniquely aligned to four major chromosomes (GWHAOSJ00000020, GWHAOSJ00000023, GWHAOSJ00000010, and GWHAOSJ00000057) (Figure 2A), indicating strong genomic coverage. A total of 32,700 expressed genes were identified, including 25,607 known genes and 7093 novel predicted genes. Additionally, 56,230 transcripts were detected, consisting of 24,627 annotated and 31,603 newly assembled transcripts. Functional annotation showed that the NR database had the highest number of gene matches, followed by the EggNOG and Pfam databases (Figure 2B). In total, 19,647 genes were successfully annotated, with 18,972 annotated in leaf samples and 17,299 shared between root and leaf tissues (Figure 2C). PCA and heatmap analysis showed a clear separation between the root and leaf samples, with tight clustering within samples, indicating high reproducibility (Figure 2D,E). The gene expression levels of the samples exhibited similar median, values further supporting data reliability (Figure 2F).

### 3.3. Co-Expression Trend Analysis of DEGs in Roots and Leaves of S. miltiorrhiza

**Figure 3:**
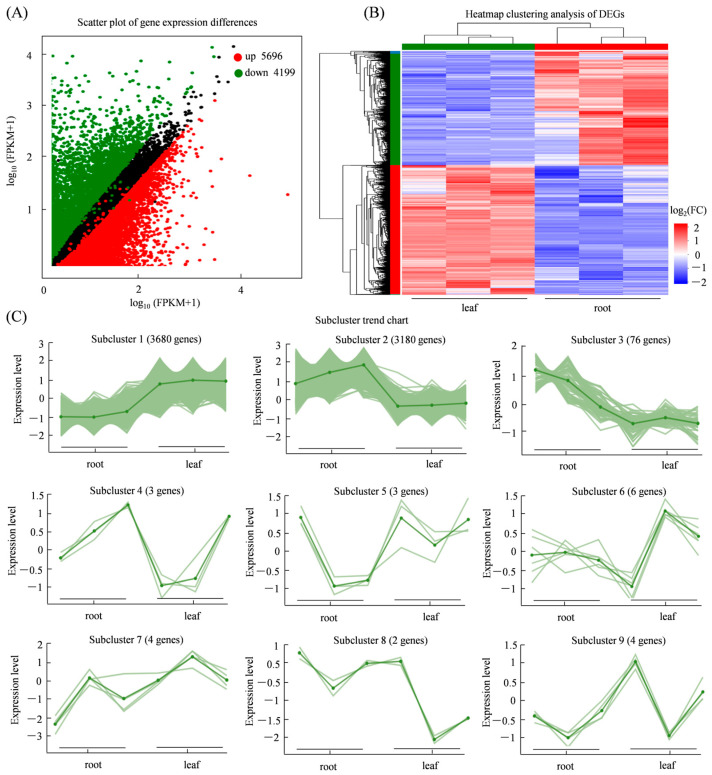
Analysis of DEGs between roots and leaves of *S. miltiorrhiza*. (**A**) Scatter plot showing upregulated (red), downregulated (blue) and non-significantly (black) differentially expressed genes; (**B**) heatmap clustering of DEGs; (**C**) co-expression trend analysis of DEGs.

A total of 9895 DEGs were identified between root and leaf tissues of *S. miltiorrhiza* (adjusted *p*-value < 0.05, |log_2_FC| ≥ 1). Among these, 5696 genes were upregulated in leaves, while 4199 were upregulated in roots (Figure 3A). Cluster analysis revealed distinct grouping patterns, with clear separation between roots and leaves, consistent with their specialized physiological functions (Figure 3B). To further explore dynamic expression patterns, a trend analysis was performed; nine distinct expression trends were identified. Among these DEGs, 3180 genes showed upregulation in roots, suggesting their potential roles in root-specific processes such as storage and biosynthesis of bioactive compounds like salvianolic acids and tanshinones (Figure 3C).

### 3.4. Functional Annotation and Enrichment Analysis of DEGs

**Figure 4:**
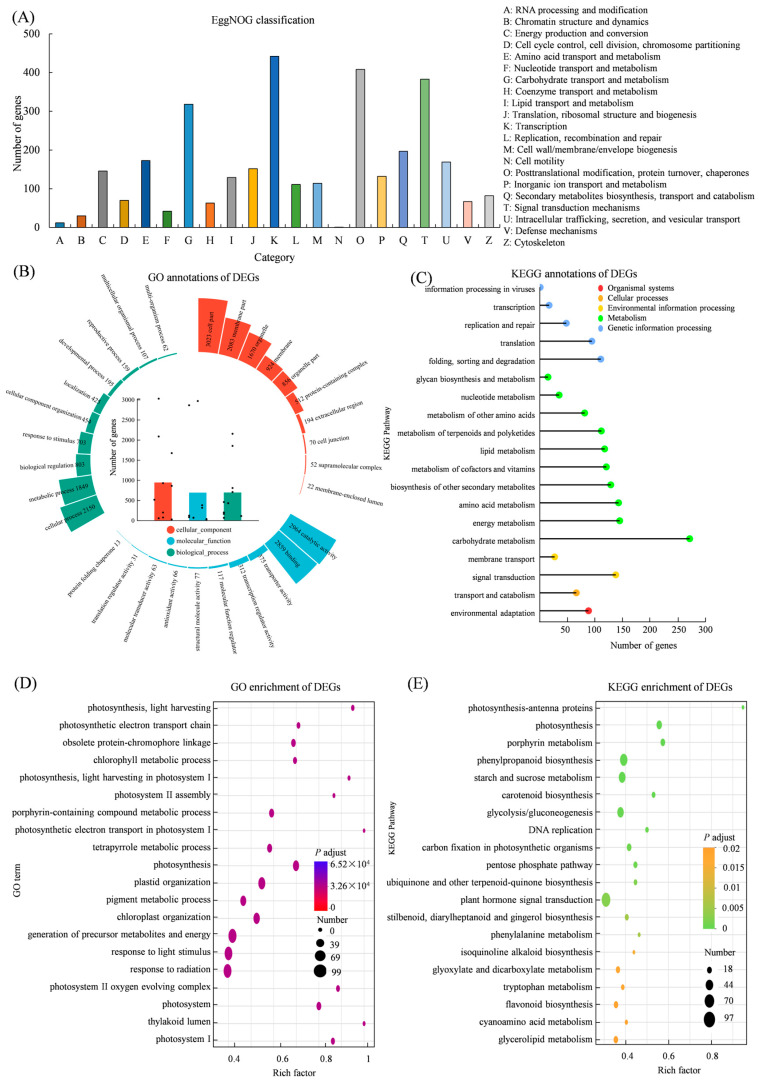
Functional annotation and enrichment of DEGs from *S. miltiorrhiza*. (**A**) EggNOG functional classification of DEGs; (**B**) GO annotation of DEGs; (**C**) KEGG pathway annotation of DEGs; (**D**) GO enrichment analysis of DEGs; (**E**) KEGG enrichment of DEGs.

The EggNOG classification assigned 442 DEGs to the category of transcriptional regulation, followed by post-translational modification, protein turnover and chaperones (408 genes), signal transduction mechanisms (383 genes), and carbohydrate transport and metabolism (318 genes) (Figure 4A). GO classification showed that within the biological process category, the most significantly represented secondary terms were cellular processes and metabolic processes. In the cellular component, the dominant annotations were cell part, membrane part, and organelle. In the molecular function category, catalytic activity and binding were the most enriched terms (Figure 4B). KEGG annotation revealed that the most enriched secondary terms in the metabolism category were associated with carbohydrate metabolism, such as carbon fixation in photosynthetic organisms, starch and sucrose metabolism, flavonoid biosynthesis, stilbenoids, diarylheptanoid and gingerol biosynthesis, and terpenoid backbone biosynthesis. In the genetic information processing category, the most annotated pathways were the MAPK signaling pathway and plant hormone signal transduction (Figure 4C). GO enrichment showed a significant number of terms related to photosynthesis, including photosynthetic electron transport in photosystem I, light harvesting in photosystem I, and photosynthesis-light harvesting (Figure 4D). KEGG enrichment analysis indicated that DEGs were significantly enriched in carotenoid biosynthesis and phenylalanine metabolism (Figure 4E).

### 3.5. Analysis of Protein–Protein Interactions and Differentially Expressed Transcription Factors

**Figure 5:**
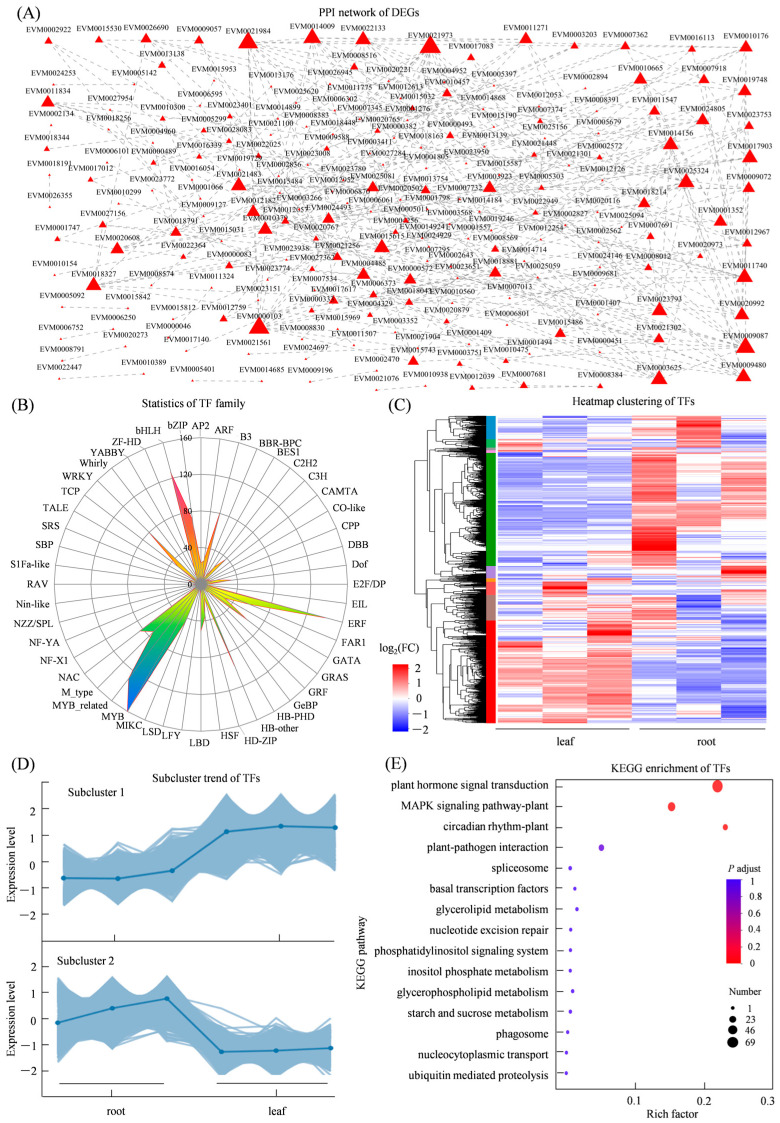
Protein interactions networks and transcription factor analyses of DEGs in roots and leaves of *S. miltiorrhiza*. (**A**) PPI network of DEGs, node size reflects interaction degree; larger nodes indicate more interactions; (**B**) statistics of differentially expressed transcription factor families; (**C**) cluster analysis of transcription factor expression patterns; (**D**) co-expression trends among transcription factors; (**E**) KEGG pathway enrichment of differentially expressed transcription factors.

The PPI network showed interactions between EVM0023151 (chromosome transmission fidelity protein 18 homolog isoform X1,CTF18) and EVM0012759 (DNA polymerase epsilon catalytic subunit A-like isoform X1,TIL1) and EVM0001407 (phytochrome interacting factor 3, PIF3) and EVM0020973 (encoding phytochrome A-like, PHYA), suggesting a potential role of light signaling and photomorphogenic regulation in tissue-specific gene expression (Figure 5A). Among the differentially expressed transcription factors, the MYB family was the most abundant, followed by ERF, bHLH, and NAC (Figure 5B). Additionally, the expression levels of these transcription factor genes exhibited two primary co-expression patterns. These TF families are known to regulate diverse biological processes including secondary metabolism and developmental transitions, which may be critical to the tissue-specific accumulation of medicinal compounds in *S. miltiorrhiza* (Figure 5C,D). KEGG enrichment analysis of the interacting proteins and associated TFs identified 15 significantly enriched pathways, the most enriched pathway was plant hormone signal transduction (map04075) and the MAPK signaling pathway (map04016) (Figure 5E).

### 3.6. Evaluation of Metabolomic Profiles in Roots and Leaves of S. miltiorrhiza

**Figure 6:**
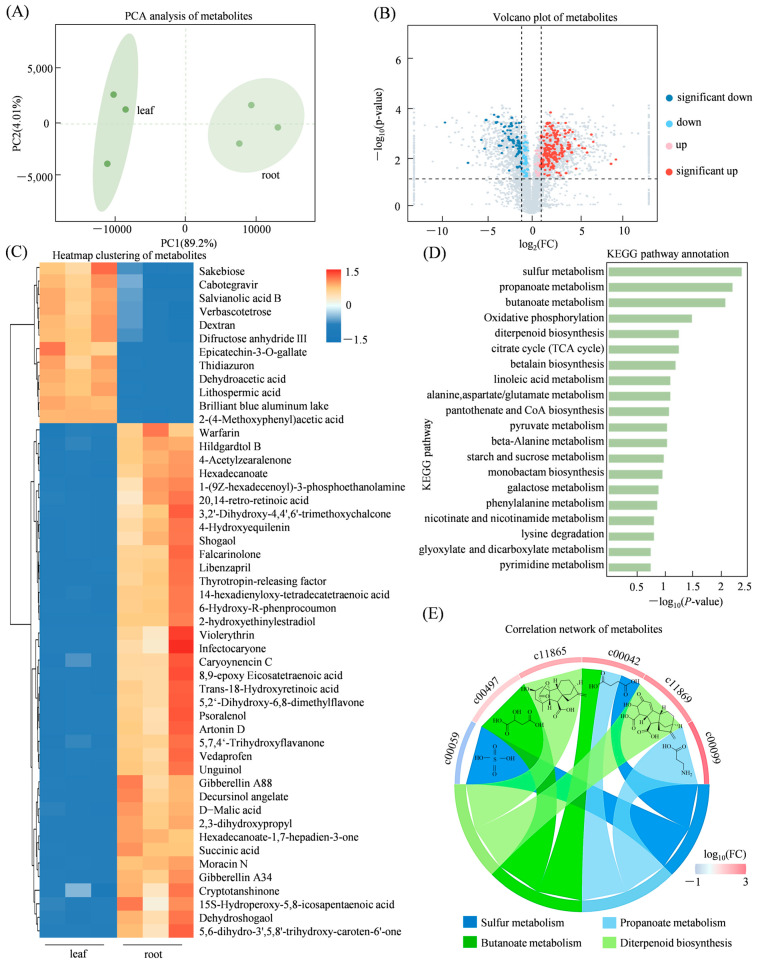
Metabolomic profiling and differential metabolite analysis of *S. miltiorrhiza* roots and leaves. (**A**) PCA score plot showing metabolic differences between tissues; (**B**) Volcano plot of significantly altered metabolites; (**C**) hierarchical clustering of differential metabolites; (**D**) KEGG pathway annotation of differential metabolite; (**E**) six major differential metabolites significantly enriched in KEGG pathways.

Broad-targeted metabolomic analysis identified a total of 6805 metabolites, among them, 241 were DEMs (172 upregulated and 69 downregulated in the root tissues compared to the leaves (Figure 6A–C). KEGG pathway enrichment analysis of the differentially accumulated metabolites indicated significant associations with multiple metabolic pathways, most notably sulfur metabolism, propanoate metabolism, butanoate metabolism, oxidative phosphorylation, and diterpenoid biosynthesis (Figure 6D). The most enriched metabolites were sulfate (C00059), D-malic acid (C00497), succinic acid (C00042), 3-aminopropionic acid (C00099), and two gibberellin derivatives: gibberellin A51 (C11865) and its catabolite gibberellin A34-catabolite (C11869) (Figure 6E).

### 3.7. Integrated Analysis of Genes and Metabolites in the Tanshinone Biosynthesis Pathway

**Figure 7:**
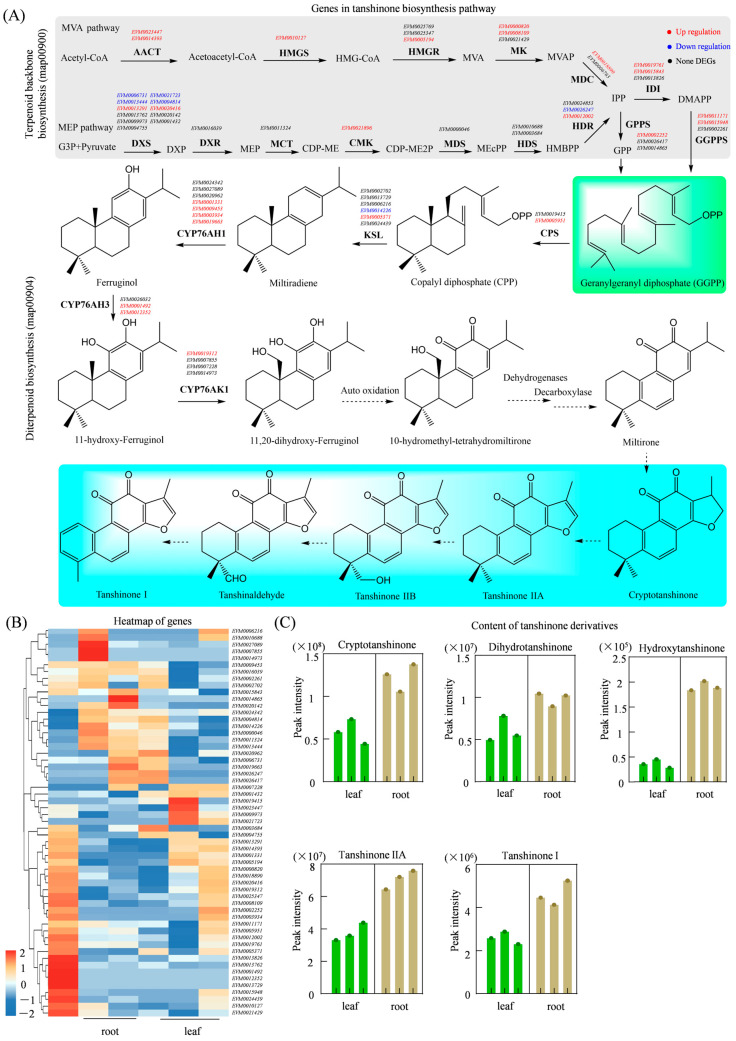
Integrated gene expression and metabolite accumulation in the tanshinone biosynthesis model of *S. miltiorrhiza*. (**A**) Expression levels of key genes involved in tanshinone biosynthesis, solid arrows indicate verified synthesis pathways, and dashed arrows indicate predicted pathways; (**B**) cluster analysis of tanshinone-related genes in roots and leaves; (**C**) quantification of tanshinone-related metabolites in roots and leaves.

Tanshinones are synthesized through the mevalonate (MVA) and methylerythritol phosphate (MEP) pathways (map00900). Within this pathway, a total of 41 genes have been identified to be associated with the production of GGPP, which is the central precursor for diterpenoid biosynthesis. Further analysis of the diterpenoid biosynthesis pathway (map00904) has revealed key putative enzymatic candidates, including two CPS, six KS, seven CYP76AH1, three CYP76AH3, and four CYP76AK1, which are known to catalyze critical steps in tanshinone formation and are putative candidates for tanshinone biosynthesis in *S. miltiorrhiza*. Of these genes, 31 have shown differential expression between roots and leaves, with the majority exhibiting significantly higher transcript levels in roots (Figure 7A,B). One notable gene, *EVM0005951*, located on chromosome GWHAOSJ00000013 and predicted to encode a terpene synthase, displayed strong root-preferential expression with a log_2_FC of 3.67. Similarly, *EVM0005371*, annotated as *ent*-kaur-16-ene synthase and located on chromosome GWHAOSJ00000023, was upregulated in roots (log_2_FC value of 1.71), suggesting its potential involvement in early diterpene skeleton formation. Several P450s implicated in the oxidation and structural diversification of tanshinone intermediates have also been identified. These include two *CYP76AH1* genes (*EVM0001331* and *EVM0009453*), two *CYP76AH3* genes (*EVM0001492* and *EVM0012352*), and one *CYP76AK1* gene (*EVM0019312*). All five of these genes showed pronounced root-specific expression, with log_2_FC values exceeding 8.43, suggesting their potential role in the late-stage modification of tanshinones. Despite comprehensive metabolite profiling, direct intermediates of the core tanshinone biosynthetic pathway (e.g., miltiradiene or ferruginol) were not detected in our samples, possibly due to their low abundance and rapid turnover in detection. However, downstream tanshinone derivatives, including dihydrotanshinone, hydroxytanshinone, cryptotanshinone, tanshinone IIA, and tanshinone I, were significantly more abundant in roots than in leaves, consistent with the observed upregulation of putative biosynthetic genes (Figure 7C). The integration of transcriptomic and metabolomic data thus highlights a suite of high-priority candidate genes that likely drive tanshinone production in *S. miltiorrhiza*.

### 3.8. Integrated Analysis of Genes and Metabolites in the Salvianolic Acid Biosynthesis Pathway

**Figure 8:**
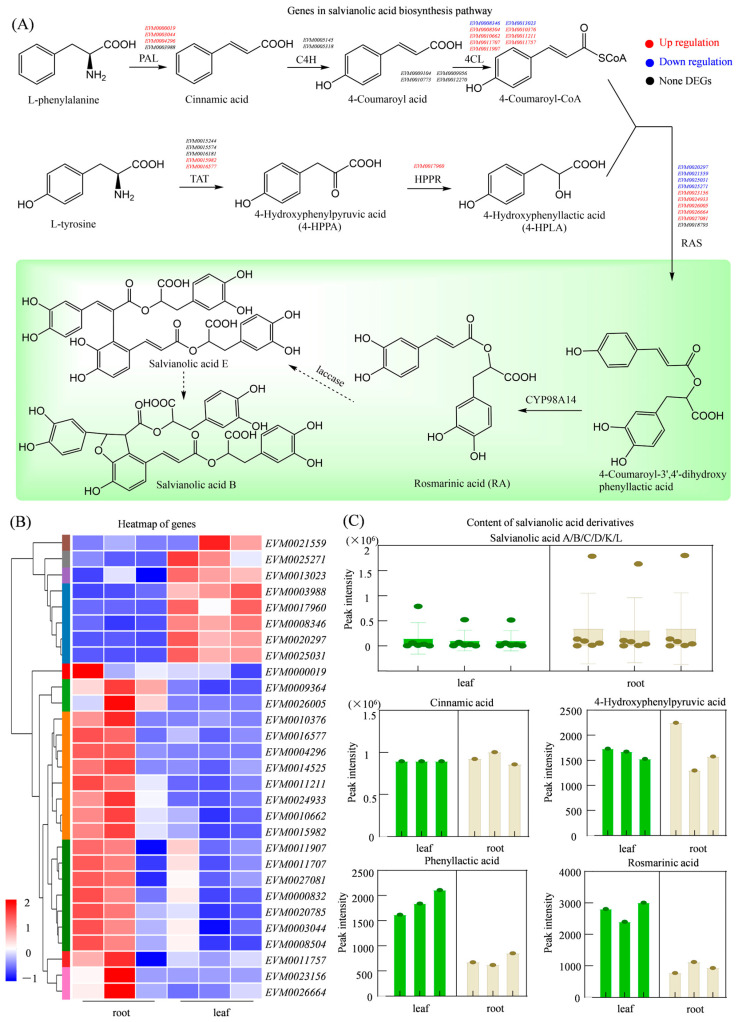
Integrated gene expression and metabolite accumulation in the salvianolic acid biosynthesis model of *S. miltiorrhiza*. (**A**) Expression levels of genes involved in salvianolic acid biosynthesis, solid arrows indicate verified synthesis pathways, and dashed arrows indicate predicted pathways; (**B**) cluster analysis of genes involved in salvianolic acid biosynthesis; (**C**) quantification of salvianolic acid-related metabolites in roots and leaves.

Salvianolic acids are important bioactive compounds found in *S. miltiorrhiza*, contributing significantly to its pharmacological properties. While the early steps of salvianolic acid biosynthesis, specifically those leading to rosemarinic acid, are relatively well characterized, the downstream pathways that give rise to complex derivatives such as salvianolic acid B remain incompletely understood. In this study, we systematically identified 39 putative candidate genes involved in the phenylpropanoid (map00940) and tyrosine metabolism (map00350) pathways, These include four *PAL* genes (*EVM0000019*, *EVM0003044*, *EVM0004296*, and *EVM0003988*), two *C4H* genes (*EVM0005145*, *EVM0005318*), thirteen *4CL* genes, five *TAT* genes, one *HPPR* gene, ten *RAS* genes, and four *CYP98A* genes. Of these, 29 genes were upregulated in root tissues, consistent with the root accumulation of salvianolic acids. Among them, three *PAL* genes (*EVM0000019*, *EVM0003044*, and *EVM0004296*), were significantly upregulated in roots, with log_2_FC values of 1.62, 3.95, and 9.24, respectively. Among the thirteen *4CL* genes, only two (*EVM0008346* and *EVM0013023*) were downregulated, while the remaining eleven showed increased expression in roots. The *RAS* genes, responsible for the formation of rosmarinic acid, displayed strong root-specific expression. Five *RAS* genes (*EVM0023156*, *EVM0024933*, *EVM0026005*, *EVM0026664*, and *EVM0027081*) were upregulated in roots, with log_2_FC values exceeding 6.15 (Figure 8A,B), suggesting their pivotal role in driving rosmarinic acid production (Figure 8C).

### 3.9. Gene Functional Enrichment and Protein–Protein Interaction Analysis in the Tanshinone and Salvianolic Acid Biosynthesis Pathway

**Figure 9:**
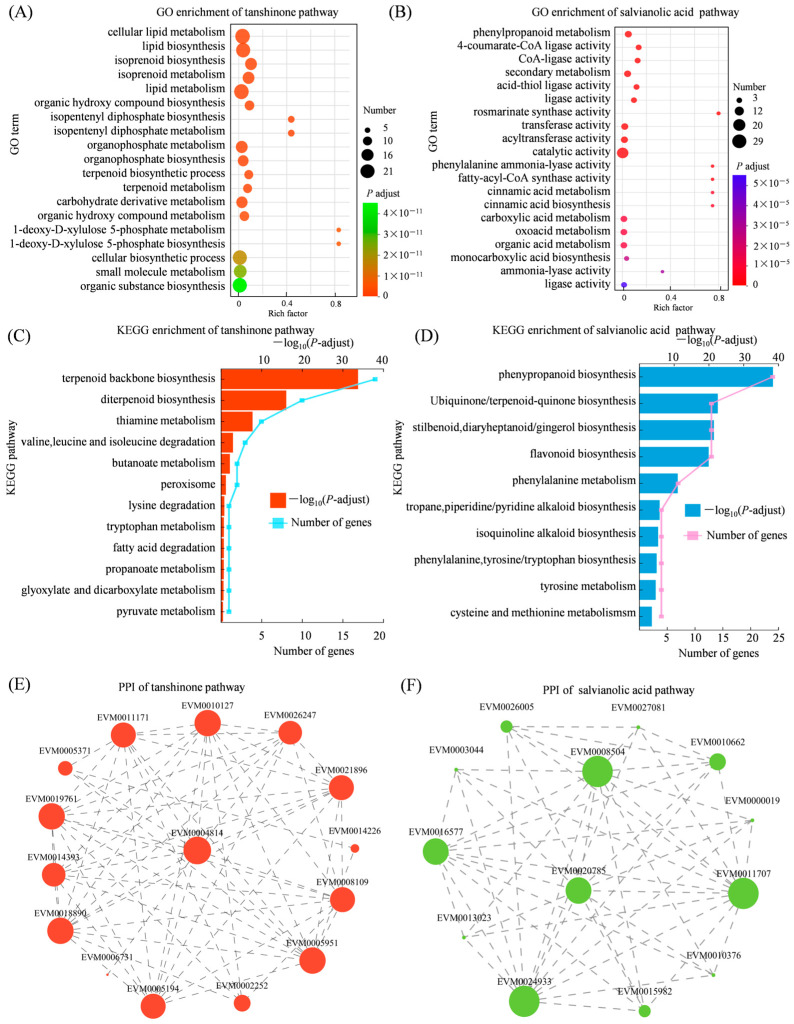
Functional enrichment and protein interactions of genes in the tanshinone and salvianolic acid biosynthesis pathways of *S. miltiorrhiza*. (**A**) GO enrichment of tanshinone biosynthesis pathway genes; (**B**) GO enrichment of salvianolic acid biosynthesis pathway genes; (**C**) KEGG enrichment of tanshinone biosynthesis pathway genes; (**D**) KEGG enrichment of salvianolic acid biosynthesis pathway genes; (**E**) PPI of proteins in the tanshinone pathway; (**F**) PPI of proteins in the salvianolic acid pathway, node size reflects interaction degree; larger nodes indicate more interactions.

The GO enrichment and KEGG pathway analyses showed that genes associated with tanshinone biosynthesis were significantly enriched in terms related to mevalonate kinase activity, 4-(cytidine 5′-diphospho)-2-C-methyl-D-erythritol kinase activity, and the metabolic process of 1-deoxy-D-xylulose 5-phosphate (Figure 9A). Salvianolic acid biosynthesis-associated genes were enriched in catalytic activities directly linked to phenylpropanoid and tyrosine metabolism; other GO terms included 2-oxoglutarate aminotransferase activity, L-tyrosine aminotransferase activity, rosmarinate synthase activity, and trans-cinnamate 4-monooxygenase activity (Figure 9B). KEGG enrichment showed that genes in the tanshinone pathway were enriched to terpenoid backbone biosynthesis, thiamine metabolism, butanoate metabolism, and pyruvate metabolism (Figure 9C). Conversely, salvianolic acid pathway genes were enriched in pathways closely tied to phenylpropanoid and specialized metabolite biosynthesis, including terpenoid-quinone biosynthesis, stilbenoids, diarylheptanoid and gingerol biosynthesis, phenylalanine metabolism, flavonoid biosynthesis, tropane, piperidine and pyridine alkaloid biosynthesis, phenylpropanoid biosynthesis, isoquinoline alkaloid biosynthesis, phenylalanine, tyrosine and tryptophan biosynthesis, tyrosine metabolism, and cysteine and methionine metabolism (Figure 9D). The computationally predicted PPI networks related to the tanshinone and salvianolic acid biosynthetic pathways were constructed; in the tanshinone biosynthesis pathway, EVM0011171 (GGPPS1) was predicted to interact with EVM0005371 (GA2), EVM0010127 (HMGS), EVM0004814 (DXS), and EVM0026247 (ISPH) (Figure 9E). In the salvianolic acid biosynthesis pathway, EVM0011707 (PAL) showed predicted interactions with EVM0015982 (4CL), EVM0026005 (TAT), and EVM0008504 (CYP73A5) (Figure 9F). These in silico-derived interactions provide potential functional associations and represent testable hypotheses that await further experimental validation (e.g., Y2H, Co-IP, or BiFC).

### 3.10. Mining of Cytochrome P450 Genes Potentially Involved in Diterpene Backbone Modification

**Figure 10:**
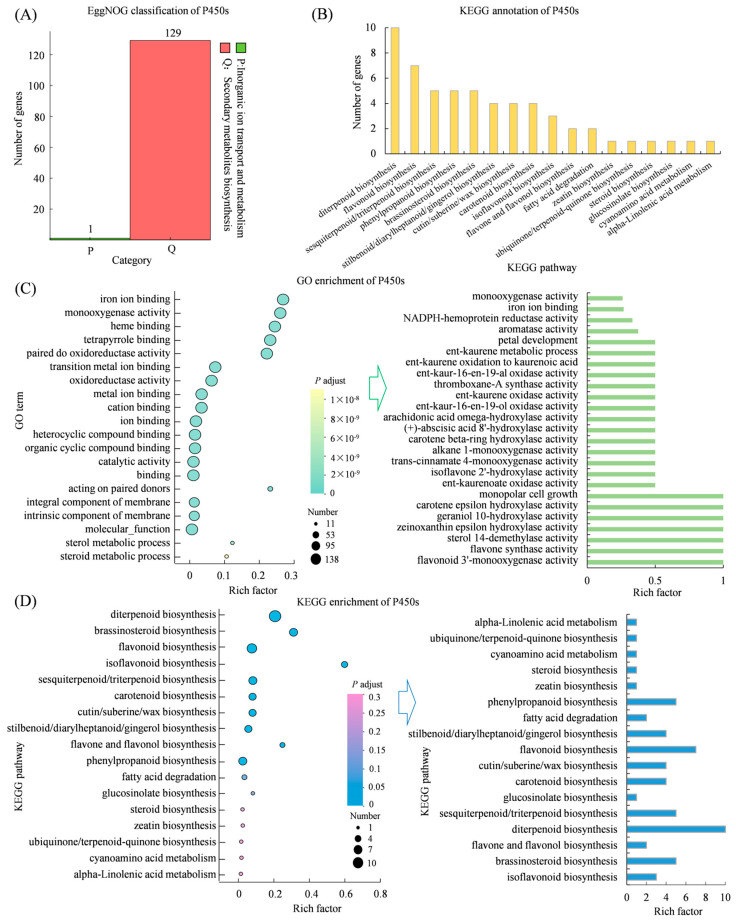

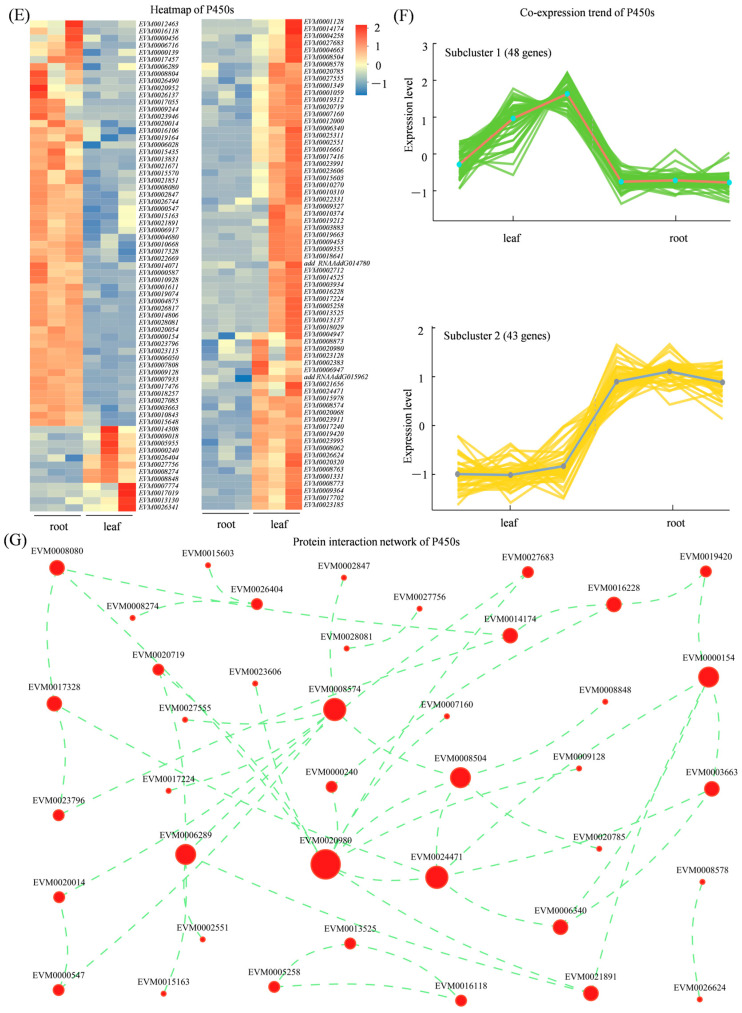
Functional characterization of candidate P450s involved in diterpene backbone modification of *S. miltiorrhiza*. (**A**) EggNOG classification of P450s; (**B**) KEGG annotation of P450s; (**C**) GO enrichment analysis of P450s; (**D**) KEGG enrichment of P450s; (**E**) Heatmap showing P450 expression patterns; (**F**) co-expression trend analysis of P450s; (**G**) protein interaction network of P450s, node size reflects interaction degree; larger nodes indicate more interactions.

A total of 138 genes annotated were as P450s in *S. miltiorrhiza*. EggNOG functional classification revealed that 129 of these genes may be associated with secondary metabolite biosynthesis, underscoring their likely role in specialized metabolism (Figure 10A). KEGG annotation mapped these P450s to 17 distinct metabolic pathways, indicating their diverse functions (Figure 10B). GO enrichment analysis showed significant enrichment in molecular functions such as iron ion binding, heme binding, and monooxygenase activity, which are key features of P450s. Specifically, these genes were associated with diverse catalytic activities relevant to plant-specialized metabolism, including flavonoid 3′-monooxygenase activity, flavone synthase activity, sterol 14-demethylase activity, zeinoxanthin epsilon hydroxylase activity, geraniol 10-hydroxylase activity, carotene epsilon hydroxylase activity, monopolar cell growth, ent-kaurenoate oxidase activity, isoflavone 2′-hydroxylase activity, trans-cinnamate 4-monooxygenase activity, alkane 1-monooxygenase activity, carotene beta-ring hydroxylase activity, (+)-abscisic acid 8′-hydroxylase activity, arachidonic acid omega-hydroxylase activity, ent-kaur-16-en-19-ol oxidase activity, ent-kaurene oxidase activity, thromboxane-A synthase activity, ent-kaur-16-en-19-al oxidase activity, ent-kaurene oxidation to kaurenoic acid, ent-kaurene metabolic process, and petal development-related functions (Figure 10C). Notably, several of these activities, particularly those involving ent-kaurene oxidation, are directly analogous to known steps in diterpenoid biosynthesis, suggesting potential functional conservation in tanshinone pathways. KEGG enrichment revealed that P450s are prominently involved in the biosynthesis of key specialized compounds, such as diterpenoid biosynthesis (10 annotated genes), flavonoid biosynthesis (7 genes), brassinosteroid biosynthesis (5 genes), sesquiterpenoid and triterpenoid biosynthesis (5 genes), and phenylpropanoid biosynthesis (5 genes) (Figure 10D). To explore spatial regulation, we analyzed the expression profiles of the 138 *P450s* in roots and leaves. Hierarchical clustering revealed two major co-expression subclusters: subcluster 1 with 48 genes preferentially expressed in roots, and subcluster 2 with 43 genes showing higher expression in leaves (Figure 10E,F). The root-preferential expression of numerous *P450s* aligns with the root-specific accumulation of tanshinones, indicating that the *P450s* in subcluster 1 may serve as candidate regulators involved in diterpenoid modification in the periderm and vascular tissues of *S. miltiorrhiza* roots, and their actual functions need validation in future study. PPI analysis showed that KAO1 (EVM0016228) interacts with KO (EVM0019420), CYP71A22 (EVM0000240), and CYP714A1 (EVM0014174), suggesting a possible multi-enzyme complex involved in progressive oxidation of the diterpene backbone from ent-kaurene to kaurenoic acid. Additionally, CYP90D1 (EVM0024471) interacts with CYP90C1 (EVM0003663), CYP72A15 (EVM0020980), and CYP72A7 (EVM0017328), proteins typically involved in steroidal and diterpenoid hydroxylation, further supporting conserved mechanisms in oxidative tailoring across different diterpenoid classes (Figure 10G).

## 4. Discussion

*S. miltiorrhiza* Bunge (Danshen) has a long history of use in traditional Chinese medicine for its medicinal properties. Its therapeutic effects are primarily attributed to two major classes of bioactive compounds: lipophilic diterpenoid tanshinones (e.g., tanshinone IIA, cryptotanshinone) and water-soluble phenolic salvianolic acids (e.g., salvianolic acid B). These pharmacologically active constituents are mainly accumulated in the underground roots, which are the primary organ for secondary metabolite biosynthesis and storage. The accumulation of these specialized metabolites is tightly regulated by both genetic programs and environmental stimuli, such as light, water stress, and hormonal signaling [25,26,27]. In this study, we conducted comparative transcriptomic and metabolomic analyses between roots and leaves of *S. miltiorrhiza*, focusing on a core subset of metabolites directly involved in tanshinone and salvianolic acid biosynthesis, to identify genes potentially associated with the biosynthesis of these core metabolites [28,29,30]. Transcriptome sequencing identified over 600 tissue-specific genes in *S. miltiorrhiza*, among them, 4199 genes were significantly upregulated in roots. This transcriptional divergence may arise from differences in the physiological function of the roots and leaves of *S. miltiorrhiza* [31,32,33]. DEG enrichment revealed significant enrichment trends in pathways such as MAPK signaling, ABC transporter, phosphatidylinositol signaling, plant hormone signal transduction, diterpene biosynthesis and phenylpropanoid metabolism, suggesting the biosynthesis of tanshinones and salvianolic acids may be associated with enzymes and multiple signaling networks that respond to environmental stresses.

Tanshinones are diterpenoids derived from GGPP, which is formed via the KEGG pathway of map00900 and map00904. In this study, we identified two *CPS*, six *KS*, seven *CYP76AH1*, three *CYP76AH3*, and four *CYP76AK1* genes, all functioning as terpene cyclization or oxidation processes. Among them, only one *CPS* gene (*EVM0005051*) was upregulated in roots, suggesting its potential involvement in producing tanshinones. Previous studies have demonstrated functional diversification among CPS in *S. miltiorrhiza* [34,35]; in this study, *EVM0005051* represents a potential target for functional validation in tanshinone biosynthesis. CYP76AH1 catalyzes a unique four-electron oxidation of miltiradiene to ferruginol and participates in tanshinone biosynthesis; in this study, we mined seven CYP76AH1 genes, all showing high expression in roots, indicating they may catalyze early oxidative steps in the biosynthesis of tanshinones [36]. Furthermore, CYP76AK2 and CYP76AK3 have been rigorously validated as essential enzymes in tanshinone biosynthesis [10,37]; here, we identified four *CYP76AK1* genes. In total, 138 *P450* genes were identified in this study, among them, ten were involved in diterpenoid biosynthesis; these candidates offer valuable resources for future investigations into the post-cyclization tailoring reactions that generate structural diversity among tanshinone.

Salvianolic acids are complex polyphenols derived from phenylalanine and tyrosine, synthesized through the convergence of the phenylpropanoid (map00940) and tyrosine metabolism (map00350) pathways. We identified 39 genes associated with these pathways, including four *PAL* genes, two *C4H* genes, and thirteen *4CL* genes, the majority of which were upregulated in roots. It is important to note that *PAL*, *C4H*, and *4CL* genes are also central to monolignin biosynthesis, and *S. miltiorrhiza* roots exhibit high levels of lignification. Since both lignin monomers (H, G, S units) and salvianolic acid precursors originate from hydroxycinnamoyl-CoA esters, there exists a metabolic competition for common intermediates [38]. Therefore, elevated expression of these genes may reflect dual roles in structural reinforcement and salvianolic acid biosynthesis. To resolve this ambiguity, future work should employ promoter–reporter assays, enzyme kinetics, and tissue-specific silencing to dissect carbon flux partitioning between lignin and salvianolic acid pathways. We mined ten *RAS* genes, among them, five genes (*EVM0023156*, *EVM0024933*, *EVM0026005*, *EVM0026664*, and *EVM0027081*) were root-upregulated, suggesting their potential involvement in the biosynthesis of salvianolic acid. Protein–protein interaction predictions revealed that, in the tanshinone pathway, GGPPS1 (EVM0011171) interacts with GA2 (ent-kaurene synthase), HMGS (HMG-CoA synthase), DXS (1-deoxy-D-xylulose-5-phosphate synthase), and ISPH (HMBPP reductase), key enzymes spanning the MEP/MVA pathways to GGPP synthesis. Also, in the salvianolic acid biosynthesis pathway, PAL (EVM0011707) interacts with 4CL, TAT, and CYP73A5 (C4H), suggesting that both the tanshinone and salvianolic acid biosynthesis pathways may operate as spatially organized complexes [39]; future validation using Y2H, Co-IP, or BiFC will be crucial to confirm physical associations.

Metabolomics analysis showed that dihydrotanshinone, hydroxytanshinone, cryptotanshinone, tanshinone IIA, and tanshinone I are significantly more enriched in roots than leaves. However, intermediate metabolites such as miltiradiene and ferruginol were detected. Interestingly, roots exhibited a higher content of phytohormones and related metabolites, such as gibberellin, which is involved in oxidative phosphorylation and diterpenoid biosynthesis. These results suggests that beyond tanshinones and salvianolic acids, other compounds may also be key pharmacological components of *S. miltiorrhiza* [8]. Moreover, the enzymatic activities of POD, SOD, CAT, and PAL were significantly higher in roots than in leaves, these enzymes contribute to redox balance and metabolism-related physiological processes, which may reflect both organ-specific basal functions and tissue-specific characteristics, rather than a generalized stress response. This further supports the important role of roots at the intersection of primary and secondary metabolism [40].

## 5. Conclusions

RNA-Seq analysis identified 2680 upregulated genes in *S. miltiorrhiza* roots, with elevated levels of POD, SOD, CAT, and PAL suggesting enhanced oxidative stress defense capacity and secondary metabolic activity in roots. Two CPS and six KS for tanshinone biosynthesis, and four PAL, two C4H, and thirteen 4CL for salvianolic acid biosynthesis were mined. Additionally, 129 *P450s* potentially involved in tanshinone and salvianolic acid biosynthesis were identified. While these DEGs are promising candidates, functional validation via enzymatic or transgenic approaches is required for further study. This study advances the understanding of tanshinone and salvianolic acid biosynthesis and provides exploratory insights and testable hypotheses that may serve as a preliminary reference for future metabolic engineering and molecular breeding. It should be acknowledged that significant knowledge gaps remain in the regulatory mechanisms underlying these biosynthetic pathways; future work should integrate multi-omics, functional validation, and spatial metabolomics to fully resolve these pathways in *S. miltiorrhiza*.

## Data Availability

The transcriptomic data supporting the findings of this study are openly available under NCBI reference number PRJNA1284027.
